# Natural Antioxidant Betanin Protects Rats from Paraquat-Induced Acute Lung Injury Interstitial Pneumonia

**DOI:** 10.1155/2015/608174

**Published:** 2015-03-12

**Authors:** Junyan Han, Deshun Ma, Miao Zhang, Xuelian Yang, Dehong Tan

**Affiliations:** ^1^College of Life Science and Engineering, Shenyang University, Shenyang 110044, China; ^2^College of Food, Shenyang Agricultural University, Shenyang 110866, China

## Abstract

The effect of betanin on a rat paraquat-induced acute lung injury (ALI) model was investigated. Paraquat was injected intraperitoneally at a single dose of 20 mg/kg body weight, and betanin (25 and 100 mg/kg/d) was orally administered 3 days before and 2 days after paraquat administration. Rats were sacrificed 24 hours after the last betanin dosage, and lung tissue and bronchoalveolar lavage fluid (BALF) were collected. In rats treated only with paraquat, extensive lung injury characteristic of ALI was observed, including histological changes, elevation of lung : body weight ratio, increased lung permeability, increased lung neutrophilia infiltration, increased malondialdehyde (MDA) and myeloperoxidase (MPO) activity, reduced superoxide dismutase (SOD) activity, reduced claudin-4 and zonula occluden-1 protein levels, increased BALF interleukin (IL-1) and tumor necrosis factor (TNF)-*α* levels, reduced BALF IL-10 levels, and increased lung nuclear factor kappa (NF-*κ*B) activity. In rats treated with betanin, paraquat-induced ALI was attenuated in a dose-dependent manner. In conclusion, our results indicate that betanin attenuates paraquat-induced ALI possibly via antioxidant and anti-inflammatory mechanisms. Thus, the potential for using betanin as an auxilliary therapy for ALI should be explored further.

## 1. Introduction

Acute lung injury (ALI) is a disease with high morbidity and mortality in the critically ill patient population [1] and is triggered by various pathogens and toxins. Oxidative stress and acute inflammation, which follow infection and trauma, are intricately involved in the development of ALI [2]. Various animal models of chemically induced ALI have been utilized to explore the pathogenesis of this disease, as well as potential therapeutic interventions.

Paraquat (1,1′-dimethyl-4,4′-bipyridinium dichloride) is a quaternary nitrogen herbicide used worldwide because of its high efficacy and low residue in crops [3]. It has been demonstrated to be highly toxic to humans and animals when absorbed via ingestion, skin contact, or inhalation. Paraquat undergoes redox cycling, being reduced by an electron donor, such as NAPDH, to form a paraquat radical, which is rapidly oxidized back to the parent compound with the concomitant transfer of the extra electron to molecular oxygen, forming superoxide anion (O_2_^∙−^), a major reactive oxygen species. Subsequently, O_2_^∙−^ is reduced to generate hydrogen peroxide (H_2_O_2_) and highly toxic hydroxyl radicals (HO^∙−^) [4]. These reactive oxygen species cause cellular damage via lipid peroxidation, activation of NF-*κ*B, mitochondrial damage, and apoptosis [5]. In the lungs, accumulation of paraquat occurs through the highly developed polyamine uptake system [3, 6], leading initially to ALI and ultimately to lung fibrosis [7]. Due to its reproducibility, simplicity of administration, and rapid effects, paraquat has been extensively used to induce ALI in experimental animal models [8, 9].

Since the mechanism by which paraquat induces its toxic effects is oxidative stress, emphasis has been placed on the use of antioxidants as a treatment for paraquat toxicity [10, 11]. The antioxidative function of natural pigments such as betalains has attracted attention in recent years [12]. Betalains are pigments present in red beetroot (*Beta vulgaris *var*. rubra*) and several other species such as amaranthus, rose, and cactus pear; they are used in food products such as processed meat, candies, and yogurt [13]. Betanin (betanidin 5-*O*-*β*-D-glucoside, red) is the most abundant betalain component, making up 75–95% of total betalains [14]. The betanin molecule includes phenolic and cyclic amine groups, which are good electron donors that endow betanin with exceptionally high free-radical scavenging ability. The results of several experiments have shown that betanin and betalains possess antioxidant and anti-inflammatory components [15, 16]. Betalains make red beetroots one of the 10 most potent antioxidant vegetables [17], which are reported to protect mice during gamma irradiation [18]. Betanin also inhibits lipid peroxidation and heme decomposition* in vitro *in extremely small concentrations [19] and attenuates carbon tetrachloride-induced liver injury in fish (*Cyprinus carpio* L.), as we have reported [20].

As such, we explored whether betanin can alleviate the oxidative stress and acute inflammation associated with ALI. To clarify the efficacy and possible mechanism of betanin on ALI, the present study investigated the effects of betanin on the pathophysiology of rat ALI induced by paraquat, including lung pathology, lung permeability, epithelial tight junctions (TJs), oxidative stress, and inflammatory cytokine levels.

## 2. Materials and Methods

### 2.1. Animals and Paraquat-Induced ALI Model

The experiment was compliant with World Medical Association Declaration of Helsinki, and the animal experiment was approved by the Animal Ethics Committee of Shenyang University.

Specific pathogen-free (SPF) grade male Sprague-Dawley rats (220 ± 20 g) were obtained from Liaoning Provincial Laboratory Animal Public Service Center (Benxi, China). Rats were housed at 22 ± 2°C under a 12-hour light–dark cycle with access to standard laboratory feed and water. After a 1-week acclimatization period, the animals were divided randomly into four groups each containing 10 rats: vehicle control group, paraquat control group, betanin 25 mg/kg per day group, and betanin 100 mg/kg per day group. Betanin (CAS number: 7659-95-2, TCI, Japan) 25 or 100 mg/kg per day as appropriate was administered by intragastric gavage (i.g., distilled water as vehicle) for five consecutive days (for 3 days before and 2 days after paraquat exposure). The rats in the vehicle control and paraquat control groups were administered the same volume of distilled water by i.g. On day 3 following administration of betanin, rats in the paraquat control, betanin 25 mg/kg per day, and betanin 100 mg/kg per day groups were intraperitoneally (i.p., normal saline as vehicle) injected with 20 mg/kg paraquat (Sigma, USA) [9]. Rats in the vehicle control group were administered an equal volume of normal saline by i.p. All animals were sacrificed 24 hours after the last betanin administration with phenobarbital sodium (i.p., 50 mg/kg) anesthesia, each animal's weight was recorded, and blood and lung samples were immediately harvested.

### 2.2. Bronchoalveolar Lavage Collection, BALF Cell Counts, and Cytokine Measurements

The lungs were weighed, and bronchoalveolar lavage fluid (BALF) was collected as previously described [21]. Total BALF cell count was determined using a hemocytometer. The BALF was centrifuged at 300 ×g for 20 min. The precipitate was stained with Wright-Giemsa dye, and a differential cell count based on morphologic criteria was carried out on 200 consecutive cells. The number of polymorphonuclear neutrophils (PMNs) in BALF was calculated by multiplying the ratio of PMNs by the total cell count [22]. Pulmonary permeability index was expressed as the ratio of protein concentration in BALF to that in plasma.

Cytokine concentrations in the BALF supernatants were determined using commercial ELISA kits (Abcam, China) specific for tumor necrosis factor- (TNF-) *α*, interleukin- (IL-) 1*β*, and IL-10.

### 2.3. Histological Examination

The left lung from each animal was fixed in the distended state by infusion of 10% formalin into the trachea and then incubated in 10% formalin for 1 week. The lung tissues were processed automatically using a Leica Microsystem tissue processor (ASP 300S, Germany), embedded in paraffin blocks, cut into 7-*μ*m slices using a Leica Microsystem microtome (Model RM 2265, Germany), and stained with hematoxylin and eosin using a Leica Microsystem autostrainer (XL, Germany). The slides were viewed under an Olympus microscope (4X-1, Japan). The following four features, that is, (1) alveolar congestion, (2) hemorrhage, (3) infiltration or aggregation of neutrophils in airspace or vessel wall, and (4) thickness of alveolar wall/hyaline membrane formation, were graded on a five-point scale as previously described [12]: 0 indicates minimal damage, 1 indicates mild damage, 2 indicates moderate damage, 3 indicates severe damage, and 4 indicates maximal damage. A total lung injury score was calculated as the sum of the four items scores.

### 2.4. Measurement of Myeloperoxidase Activity, Superoxide Dismutase Activity, and Malondialdehyde Levels

The right lung of each animal was homogenized (10%, w/v) in ice-cold 1.15% KCl, 0.01 M sodium, and potassium phosphate buffer solution (pH 7.4) using a glass homogenizer. The homogenate was centrifuged at 10 000 ×g for 20 min at 4°C, and the supernatant was collected. Myeloperoxidase (MPO) activity was determined by the method of Goldblum and Jay [23], superoxide dismutase (SOD) activity was measured according to the photochemical method [24], and the malondialdehyde (MDA) level was determined as described previously [25].

### 2.5. Western Blot Analysis

Western blotting was performed as previously described [26]. Briefly, approximately 20 *μ*g of each protein sample was resolved by 10% sodium dodecyl sulfate polyacrylamide gel electrophoresis (SDS-PAGE) and transferred to polyvinylidene difluoride membranes. Membranes were incubated in tris-buffered saline (T-TBS) containing 3% non-fat dry milk with the appropriate primary antibody specific for zonula occluden- (ZO-) 1, claudin-4, or *β*-actin (Cell Signaling Technology, USA) overnight at 4°C. The blot was washed and incubated with goat anti-mouse IgG conjugated to peroxidase (Cell Signaling Technology). Antibody binding was detected by chemoluminescence staining using an ECL detection kit (Bio-Rad, Hercules, USA), and the density of each band was quantified using the Gel Doc XR system (Bio-Rad).

### 2.6. NF-*κ*B Activity Assay

Nuclear protein was extracted from lung tissue as previously described [27]. NF-*κ*B (p65) DNA-binding activity was assessed using a commercial ELISA kit (Cell Signaling Technology) following the manufacturer's protocol.

### 2.7. Protein Concentration Determination

Protein concentration in all samples was determined by the method of Bradford [28], using bovine serum albumin as the standard protein.

### 2.8. Statistical Analysis

Data were statistically assessed by analysis of variance using SPSS (IBM, Armonk, NY, USA), followed by Student Newman Keul's post hoc test. The data were expressed as mean ± SD; *P* < 0.05 was considered statistically significant.

## 3. Results

### 3.1. Effects of Betanin on Lung : Body Weight Ratio

**Figure 1:**
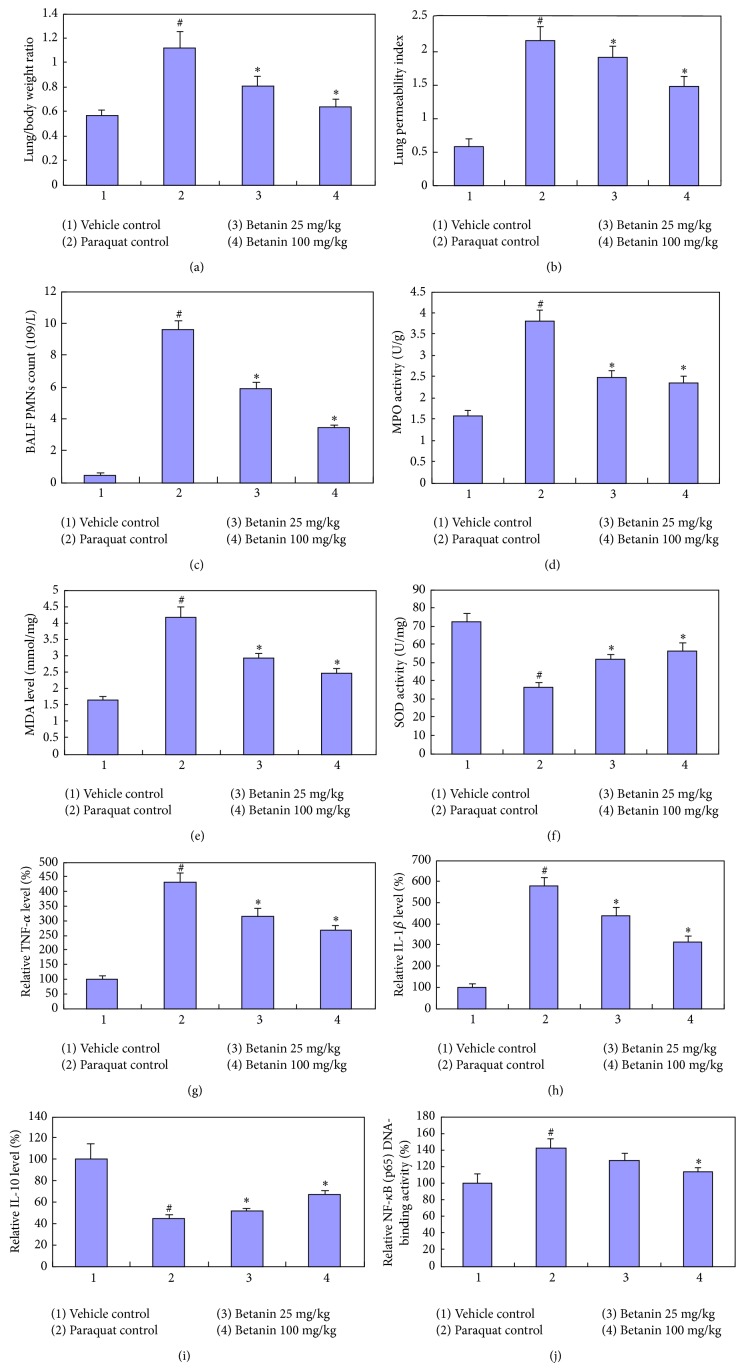
Influences of betanin on paraquat-induced rat lung parameter changes. (a) Lung/body weight ratio, (b) lung permeability index, (c) BALF PMNs count, (d) MPO activity, (e) SOD activity, (f) MDA level, (g) TNF-*α* level, (h) IL-1*β* level, (i) IL-10 level, and (j) NF-*κ*B (p65) DNA-binding activity. Paraquat was injected intraperitoneally at a single dose of 20 mg/kg body weight, and betanin (25 and 100 mg/kg/d) was orally administered 3 days before and 2 days after paraquat administration. Rats were sacrificed 24 hours after the last betanin dosage. ^#^ *P* < 0.05, compared to vehicle control; ^*^ *P* < 0.05, compared to paraquat control.

At the end of the experiment, the animals in the vehicle control group were in a good state, exhibiting normal breathing, quickness in movement, and healthy-looking fur. In contrast, cough, marked polypnea, dullness, and fluffing and withering of the fur were observed in paraquat control group rats. Animals in the two betanin groups had a better physical appearance than those in the paraquat control group. Furthermore, the lungs from rats in the paraquat control group were obviously swollen and congested. The lung : body weight ratios of the animals in the paraquat control group were significantly higher than those in the vehicle control group. In comparison, lung swelling and congestion were improved in the betanin groups, and the lung : body weight ratios of animals in the betanin groups were significantly lower than those of the animals in the paraquat control group (*P* < 0.05) (Figure 1(a)).

### 3.2. Effects of Betanin on Paraquat-Induced Changes in Lung Permeability and Lung Neutrophil Infiltration

Exposure of rats to paraquat resulted in a significant increase in lung permeability, indicating damage to the alveolar epithelial barrier (Figure 1(b)). Additionally, we observed an increase in the number of BALF PMNs in the paraquat control group compared to the number of BALF PMNs in animals from the vehicle control group (Figure 1(c)), suggesting increased cellular inflammatory infiltration into the lungs. In the betanin groups, both lung permeability and BALF PMNs were significantly lower than those in the paraquat control group, and these reductions were more pronounced in the betanin group administered 100 mg/kg betanin.

### 3.3. Effects of Betanin on Lung Histology

**Figure 2:**
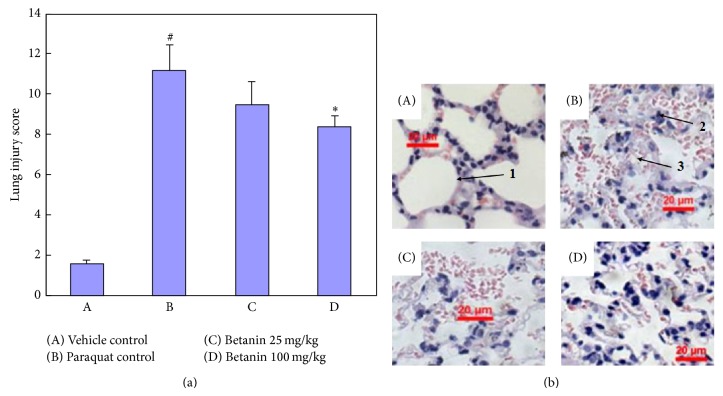
Representative lung histological pictures (b) and statistic data of injury score (a). (A) Vehicle control, (B) paraquat control, (C) betanin 25 mg/kg, and (D) betanin 100 mg/kg. Arrows indicate 1. normal alveoli, 2. thickening of interalveolar septa, and 3. hemorrhage and cellular inflammatory infiltrates. Scale bar: 20 *μ*m. ^#^ *P* < 0.05, compared to vehicle control; ^*^ *P* < 0.05, compared to paraquat control.

The left lung of each animal was fixed, sectioned, and examined for lung injury using a previously described scoring system, as detailed in Section 2. Lungs from the vehicle control group showed no lung injury, with samples demonstrating simple columnar epithelium or cuboidal epithelium respiratory bronchioles, and regular wall structures in the pulmonary alveoli. In contrast, extensive lung injury was observed in animals from the paraquat control group. Pulmonary edema, hemorrhage, cellular inflammatory infiltrates, and thickening of alveolar walls with a disorganized alveolar structure and accumulated exudates were observed in lungs of animals from the paraquat control group. The paraquat-induced lung histological changes were attenuated in the betanin treatment groups, especially in the group that received 100 mg/kg betanin. Although histological changes were still apparent, edema, hemorrhage, cellular inflammatory infiltrates, and thickening of the alveolar septa were all less than the changes observed in the paraquat control group (Figure 2). The pathological lung injury score was much higher in the paraquat control group than in the vehicle control group, and the score was less in the betanin groups than in the paraquat control group (*P* < 0.05). The effect of betanin on lung injury was observed in a dose-dependent manner.

### 3.4. Effects of Paraquat and Betanin on Tight Junction Proteins

**Figure 3:**
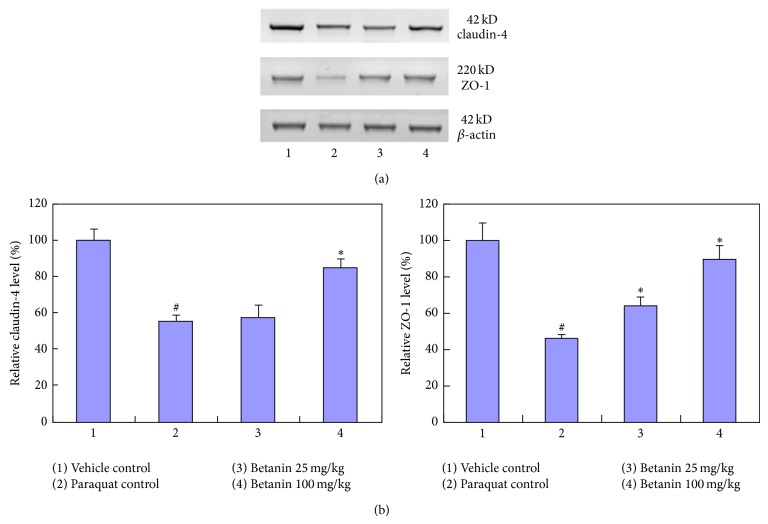
Effects of betanin treatment on lung epithelium tight junctions injury caused by paraquat. Western blots were performed to detect the abundance of tight junction proteins of claudin-4 and ZO-1. All proteins were normalized to the corresponding *β*-actin. (a): western blot images. (b): statistic data. ^#^ *P* < 0.05, compared to vehicle control; ^*^ *P* < 0.05, compared to paraquat control.

Lung protein was assessed by quantitative immunoblotting for the expression of ZO-1 and claudin-4, proteins associated with epithelial tight junctions. ZO-1 and claudin-4 protein levels were significantly lower in lung tissue from the paraquat control group compared to the levels observed in the vehicle control group. In lung tissue from both betanin groups, the paraquat-induced decrease in ZO-1 and claudin-4 protein levels was reversed in a dose-dependent manner (*P* < 0.05, Figure 3).

### 3.5. Effects of Betanin on Paraquat-Induced Oxidative Stress, Neutrophilic Inflammation, and Antioxidants

To assess the effect of betanin on paraquat-induced oxidative stress, we assessed MDA levels (a marker of oxidative stress) and SOD activity (an antioxidant status marker), while MPO levels were examined to determine the effect of betanin on paraquat-induced neutrophilic inflammation. The highest MPO activity (Figure 1(d)) and MDA level (Figure 1(e)) were observed in lung tissue from the paraquat control group. The paraquat-induced increase in MPO activity and MDA levels was significantly inhibited in rats that received 25 or 100 mg/kg betanin (*P* < 0.05). In contrast, rats in the paraquat control group showed the lowest SOD activity (Figure 1(f)), while both 25 and 100 mg/kg betanin significantly antagonized the decrease in SOD activity induced by paraquat (*P* < 0.05).

### 3.6. Effects of Betanin on Paraquat-Induced Changes in Cytokine Production and NF-*κ*B Activity

The levels of the proinflammatory cytokines IL-1*β* and TNF-*α* as well as the anti-inflammatory cytokine IL-10 were assessed in the BALF supernatants from all rats. Further, as cytokine production is tightly linked to the activation of NF-*κ*B, we assessed NF-*κ*B activity in the lung tissue using an NF-*κ*B (p65) DNA-binding activity assay. In animals from the paraquat control group, protein levels of IL-1*β* and TNF-*α* in the BALF were significantly higher than the levels observed in the control vehicle group, while the IL-10 level in the BALF was significantly lower in the paraquat control group compared to the levels of IL-10 observed in the vehicle control group. The paraquat-induced increase in the levels of TNF-*α* (Figure 1(g)) and IL-1*β* (Figure 1(h)) and the paraquat-induced decrease in IL-10 levels (Figure 1(i)) were significantly attenuated in animals in both betanin groups, and this was observed in a dose-dependent manner (*P* < 0.05).

In the paraquat control group, NF-*κ*B (p65) DNA-binding activity (Figure 1(j)) in the lung tissue was significantly higher than the activity measured in the lung tissue from the control vehicle control group. In animals administered 100 mg/kg betanin, the paraquat-induced increase in NF-*κ*B (p65) DNA-binding activity was attenuated in a dose-dependent manner (*P* < 0.05).

## 4. Discussion

Paraquat is an electron acceptor, which following reduction rapidly donates an electron to oxygen to form the superoxide anion and a series of more toxic reactive oxygen species. As such, exposure to paraquat leads to acute oxidative stress-related insult resulting in ALI [9]. Experimental paraquat-induced ALI is a well-established animal model of ALI, characterized by interstitial pneumonitis with alveolar epithelial cell and Clara cell disruption, diffuse alveolar collapse, hemorrhage, edema, hypoxemia, and infiltration of inflammatory cells into the interstitial and alveoli spaces [7, 8, 29]. In this study, paraquat administration induced the hallmarks of ALI, including interstitial pneumonitis, increased lung : body weight ratio (reflecting pulmonary edema), elevated lung permeability, and infiltration of inflammatory cells into the alveolar spaces. Interestingly, we found that betanin protected against paraquat-induced lung injury in this model of ALI.

The primary target of paraquat toxicity in the lung is the alveolar epithelium. The lung epithelium serves as a barrier forming tight junctions (TJs), which seal off the paracellular space and regulate the passage of ions and macromolecules through the paracellular pathway. TJs contain a series of interacting proteins and receptors, including zonula occludens (ZOs) 1–3, claudins 1–5, occludin, and transmembrane adhesion proteins, which usually interact in a homophilic manner [30, 31]. Decreased expression of TJs such as ZO-1 and claudin-4 has been found to play a key role in multiple epithelial and endothelial injuries characterized by high permeability [32, 33]. In this study, the paraquat-induced decrease in ZO-1 and claudin-4 indicates that paraquat damages epithelial TJs. This data is consistent with our observation of paraquat-induced increases in lung permeability and inflammation. In our model of paraquat-induced ALI, we demonstrated that betanin attenuated the ability of paraquat to reduce the expression of ZO-1 and claudin-4, thus confirming that betanin protects the barrier function of the alveolar epithelium.

To further explore the protective mechanisms of betanin, we assessed changes in the oxidative stress status of the lungs. To evaluate oxidative stress in vivo, ROS-induced modifications of cellular constituents such as lipid peroxide (MDA) and changes in antioxidative systems such as SOD are commonly assessed [34]. MPO, which is produced mainly by PMN leukocytes and as such reflects the degree of neutrophilic infiltration into tissues, is also a source of ROS, due to its ability to reduce ferritin (Fe^3+^) to free Fe^2+^, thus contributing to oxidative damage [35]. In this study, consistent with previous reports, paraquat-induced oxidative stress was demonstrated by an increase in MDA levels and MPO activity and a reduction in SOD activity [11, 36]. Betanin attenuated the paraquat-induced oxidative stress in our model, which is in agreement with its antioxidant and anti-inflammation effects [16, 18, 37].

Overproduction of ROS within the lungs can lead to oxidative stress, resulting in lung damage, and, in addition, it can induce inflammation of the airways, thus contributing to the pathogenesis of airway disease [38]. Several proinflammatory cytokines, including TNF-*α* and IL-1*β*, as well as the transcription factor NF-*κ*B play important roles in the development of lung inflammation [39]. NF-кB is essential in orchestrating the inflammatory response to a wide range of insults, including oxidative stress as well as numerous other proinflammatory stimuli. Activation of NF-кB results in the coordinated expression of inflammatory and innate immune genes as well as the secretion of inflammatory chemokines and cytokines [40]. TNF-*α* is a critical cytokine in the inflammatory and fibrotic responses in the lung following toxicant exposure [41] and has the ability to induce cell death and trigger alveolar epithelial dysfunction in acute lung injury [42]. IL-1*β* is a key mediator of the inflammatory response, being involved in a variety of cellular activities, including cell proliferation, differentiation, and apoptosis [43]. Paraquat has previously been reported to induce the expression of various inflammation cytokines, including TNF-*α* and IL-1*β*, and activate NF-кB [44–47]. This is consistent with the data presented in this report. Here, we report that betanin antagonized paraquat-induced IL-1*β* and TNF-*α* expression and attenuated paraquat-induced NF-кB activation. To our knowledge, this is the first report to confirm the anti-inflammatory role of betanin in an animal model of ALI.

In conclusion, betanin attenuates paraquat-induced ALI, and we believe that the antioxidant and anti-inflammatory properties of betanin may be involved in protecting the lung from paraquat-induced injury.

## Abbreviations

ALI: Acute lung injury
BALF: Bronchoalveolar lavage fluid
MDA: Malondialdehyde
MPO: Myeloperoxidase
SOD: Superoxide dismutase
IL-1: Interleukin
(TNF-) *α*: Tumor necrosis factor
NF-кB: Nuclear factor kappa
O_2_^∙−^: Superoxide anion
H_2_O_2_: Hydrogen peroxide
TJs: Tight junctions
SPF: Specific pathogen-free
i.p.: Intraperitoneally
PMNs: Polymorphonuclear neutrophils
SDS-PAGE: Polyacrylamide gel electrophoresis
T-TBS: Tris-buffered saline
(ZO-) 1: Zonula occluden.
